# Association of body composition with pubertal timing in children and adolescents from Guangzhou, China

**DOI:** 10.3389/fpubh.2022.943886

**Published:** 2022-08-17

**Authors:** Yijin Zheng, Jianping Liang, Ding Zeng, Weiqing Tan, Lun Yang, Shuang Lu, Wanwen Yao, Yi Yang, Li Liu

**Affiliations:** ^1^Department of Epidemiology and Biostatistics, School of Public Health, Guangdong Pharmaceutical University, Guangzhou, China; ^2^Guangzhou Health Care Promotion Center for Primary and Middle Schools, Guangzhou, China

**Keywords:** fat mass, fat-free mass, pubertal timing, children and adolescents, propensity score

## Abstract

**Objective:**

An ongoing debate surrounds the relationship between body composition and pubertal timing, in particular for boys. This cross-sectional study aimed to investigate the association of body composition with pubertal timing among children and adolescents.

**Methods:**

A total of 1,493 boys and 1,261 girls who entered puberty were enrolled in Guangzhou, China. Tanner stages were evaluated by examination of breast development for girls and testicular volume for boys. Fat mass (FM) and fat-free mass (FFM) were determined by bioelectrical impedance analysis. Parameters for body composition were transformed into age-and gender-specific *Z*-scores. The association of body composition with pubertal timing was examined using multinomial logistic regression with inverse probability weighting (IPW) based on the propensity score.

**Results:**

For boys, IPW analysis showed *Z*-scores of body fat percentage (BF%) and FM index (FMI) were negatively associated with early puberty (OR = 0.75, 95% CI = 0.64–0.87; OR = 0.74, 95% CI = 0.63–0.88). As for girls, in contrast to boys, positive associations were seen between BF% and FMI with early puberty (OR = 1.39, 95% CI = 1.19–1.64; OR = 1.59, 95% CI = 1.33–1.90). With respect to appendicular skeletal muscle mass index (ASMI), there was a positive association with early puberty and a negative one with late puberty in boys (OR = 1.26, 95% CI = 1.07–1.49; OR = 0.82, 95% CI = 0.69–0.99).

**Conclusion:**

There is a positive association of FM with early puberty for girls while negative for boys. FFM yields a positive association with early puberty and a negative one with late puberty in boys, but not in girls. Our findings highlight the gender differences in the connection between body composition and pubertal onset.

## Introduction

Puberty is an important transition phase marked by rapid physical and psychological changes from childhood to adulthood. The first landmark of pubertal events is the onset of puberty, of which the earliest external sign is the appearance of breast buds in girls and testicular enlargement in boys (1). The features of pubertal development are in a similar sequence for all children. However, age and pubertal status are partly dissociable, with a wide between-individual variation in the timing of pubertal onset. Girls normally enter puberty at ages ranging from 8 to 13 years, while boys occur ~1 year later (2). Since the late 19th century, a worldwide secular trend toward an earlier onset of puberty has been observed among both boys and girls (3–5). Beyond immediate impacts on physical and physiologic development (6–8), early onset of puberty has been broadly connected with adverse long-term adult health consequences, including increased risks of obesity, type 2 diabetes, cardiovascular diseases, and cancers (9–13). On the other hand, late puberty has a potentially negative effect on psychosocial stress and could also raise the risks of conditions, such as coronary heart disease for females, asthma for males, and poor overall health for both genders (9, 14–16). The onset of puberty is a complex and largely unknown process governed by interactions between genes and the environment (17). Identification of modifiable risk factors and underlying mechanisms for variation in puberty timing could provide useful insights to inform preventive measures to reduce the hazard.

In the 1970s, the “critical weight” hypothesis proposed by Frisch suggested increment in fat accretion is essential for triggering the puberty spurt (18). The relationship between obesity and pubertal onset has received substantial focus in recent decades (19–23). Association between obesity and early puberty has been well-established in girls (19–23), but less clear in boys. While some studies found obesity was linked to earlier puberty in boys (22, 24, 25), others reported a null association (19, 26), and yet others observed a positive association with late puberty (21, 23, 27).

The inconsistent findings in boys across studies could be due to the following reasons. (1) Reliable measure of pubertal events was challenging in boys, with different measures leading to diverse conclusions. Most studies applied the self-reported puberty questionnaires for voice break, pubic hair, growth spurt, or genital development (21, 28–30), which were susceptible to reporting bias. By contrast, examination of testicular volume is a more objective and stable assessment of the pubertal stage (31). (2) The limitation of body mass index (BMI) as a proxy of adiposity in most research might have also accounted for the inconsistency. BMI is actually a crude measure of body weight status that does not discriminate between fat mass (FM) and fat-free mass (FFM) (32). Especially in boys, weight gain is mainly structured by the increment of FFM, and thus using BMI might increase bias in the link of obesity to puberty. As BMI does not reflect the true picture of obesity, increasing attention has been attracted to the measure of body composition. Direct measurement of body fat could be obtained using bioelectrical impedance analysis (BIA), which determines the electrical impedance through the entire body and calculates estimates of FM and FFM. BIA technology has evolved remarkably, making it the most cost-effective method of measuring body composition scans (33). (3) In addition to FM, FFM might also have an impact on pubertal timing (34, 35), but with insufficient evidence on this issue. FFM and FM confer different health outcomes. Exploring the potential associations of FFM with puberty timing might be helpful to better understand the mechanism of pubertal onset. (4) Furthermore, confounding bias might constitute the main source of the inconsistencies across studies. Multiple factors, such as social and economic factors, physical activity, sleep, and dietary habit, might obscure the relationship between obesity and puberty timing (36–39). To overcome the problem of confounding bias, the propensity score-based inverse probability weighting (IPW) approach has been proposed recently (40). As an alternative method of covariate adjustment, propensity score-based IPW creates a pseudo-population in which covariate distribution is balanced between groups and mimics a randomization procedure, consequently controlling confounders in observational data (41). The propensity score-based IPW method has been reported to offer an advantage over traditional methods in minimizing the likelihood of confounding by a number of authors (42–44).

This cross-sectional study was designed to systematically investigate the association of FM and FFM with pubertal timing. In 2,754 children and adolescents (1,493 boys and 1261 girls) from Guangzhou, China, we used the segmental multi-frequency BIA to obtain body composition measures, evaluated tanner stage using testicular volume for boys and breast development for girls, and then introduced IPW based on propensity score to balance covariates and reduce confounding bias in effect estimation of body composition on pubertal timing.

## Materials and methods

### Study population

**Figure 1:**
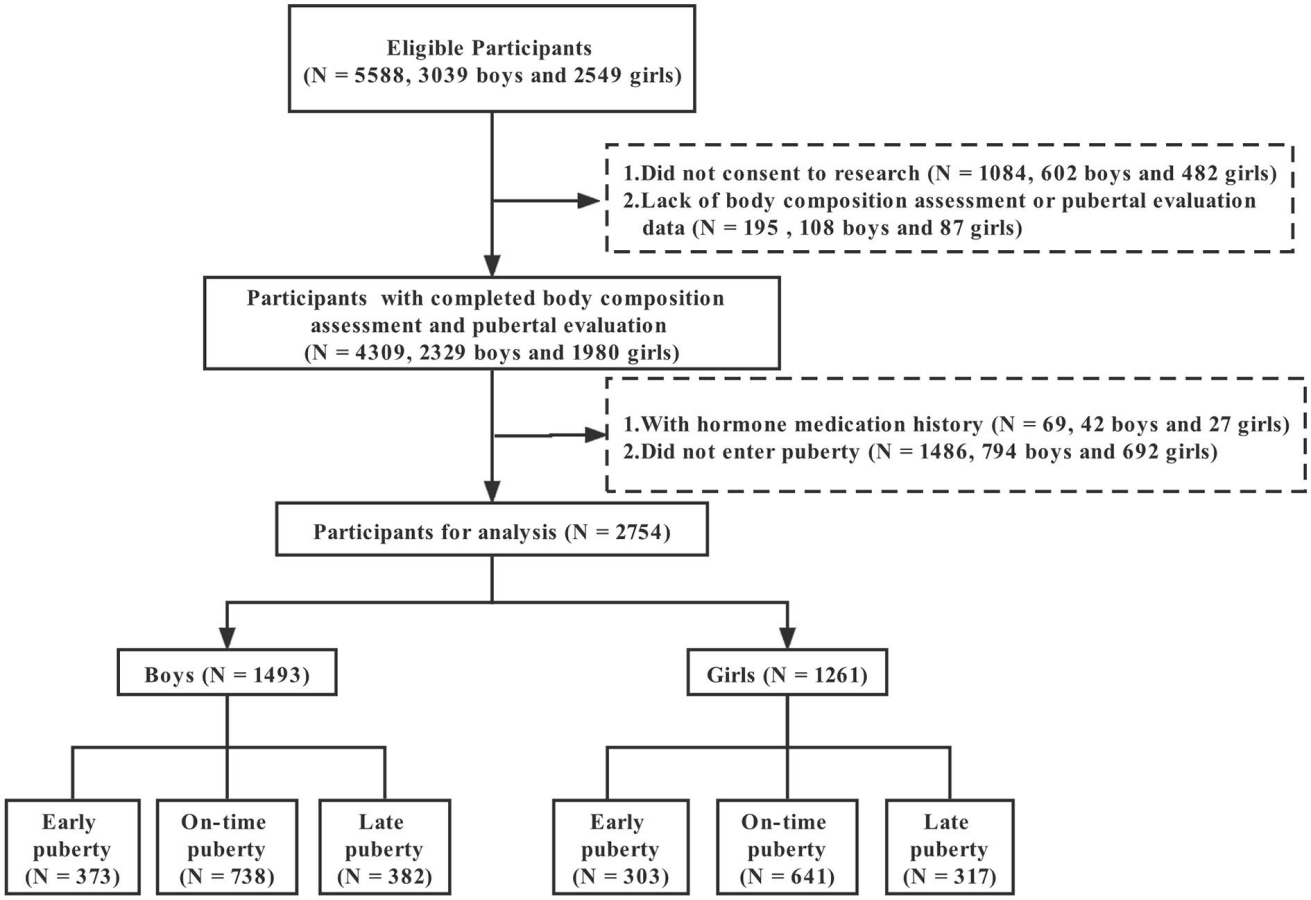
Flow diagram of study recruitment and retention.

This cross-sectional study was conducted from September 2019 to May 2020 in Guangzhou, China, with a multistage stratified cluster sampling method. The 11 administrative districts in Guangzhou were classified into three stratums according to the Gross Domestic Product (GDP) per capita in 2018. Within each stratum, one district was randomly selected. One elementary school and one middle school were then chosen randomly from each district. All students in grades 2–4 of primary schools and grades 7–8 of secondary schools were recruited with an invitation letter detailing the research objectives and potential benefits and risks of participation. Figure 1 presents the flow of study participants. A total of 5,588 students, including 3,039 boys and 2,549 girls, were invited to participate, and 2,329 boys and 1,980 girls attended and completed body composition assessment and pubertal evaluation, with response rates of 76.64 and 77.68% for boys and girls, respectively. After excluding the students with a history of hormone therapy (42 boys and 27 girls) or without entering puberty (794 boys and 692 girls), a total of 1,493 boys and 1,261 girls were finally included. All participants and their guardians provided written informed consent. The study was approved by the Ethics Review Committee of Guangdong Pharmaceutical University, China (Medical Ethics Review No.: 2018-27).

### Measurements

#### Anthropometry and body composition

All measurements were obtained by trained researchers. Anthropometric measures were taken on standing participants wearing light clothes and without shoes. Height was measured with a portable stadiometer (Seca-213, SECA, Hamburg, Germany) to the nearest 0.1 cm. Body composition was assessed by segmental multi-frequency BIA using the octopolar Inbody 570 device (Inbody Co., Ltd., Korea). Participants, wearing light clothing, stood barefoot on the metal footplates and held the hand electrodes. Outputs from the device were body impedance, body weight, and predicted measurements *via* the manufacturer's internal equations, including regional and total body FFM, FM, muscle mass (MM), etc. Weight, FM, FFM, and MM were recorded to the nearest 0.1 kg.

BF% and FFM percentage (FFM%) were calculated as (FM/weight) × 100% and (FFM/weight) × 100%, respectively. Appendicular skeletal muscle mass was calculated as the sum of muscle mass of the arms and legs. FM index (FMI), FFM index (FFMI), and appendicular skeletal muscle mass index (ASMI) were calculated as corresponding body composition parameters (kg) divided by the square of the height (m^2^). To account for differences in body composition by age and gender, we transformed age- and gender-specific Z-scores for BF%, FMI, FFM%, FFMI, and ASMI.

#### Timing of puberty

Pubertal stage was assessed by Tanner criteria of breast development (B1-B5) in girls and genital development (G1-G5) in boys (45). Two trained researchers, gender-matched to the participant, conducted the assessment under standardized conditions. Breast development was measured by visual inspection and palpation in girls, and testicular volume using a set of orchidometer beads in boys. Pubertal onset in girls was defined as progression to breast Tanner stage 2 (B2) and in boys as progression to a testicular volume of 4 ml (G2) (46, 47). After excluding prepubertal participants (Tanner stage 1), pubertal participants were classified into early (≤25th percentile of age for each stage), on-time (25th−75th percentile of age for each stage), and late pubertal timing (>75th percentile of age for each stage) (48).

#### Other covariates

A self-developed, standard questionnaire was applied to collect information about demographic characteristics (age, gender, family income, and parental education level), life behavior (physical activity, sedentary time, and sleep duration), dietary habits (intake frequency of meat, aquatic products, vegetables, bean products, fruits, egg, and milk), and history of disease and therapy. Parents completed the questionnaire of students in second–third grades, while students in higher grades completed the questionnaire themselves.

### Statistical analyses

All statistical analyses were conducted for girls and boys separately in R software (version 4.0.2). Normally distributed variables were described as mean ± standard deviation (SD), non-normally distributed continuous variables as the median and interquartile range (IQR), and categorical variables as frequency and percentage. Further analyses were performed on age- and gender-specific *Z*-score data of body composition. One-way ANOVA analysis was used to determine the differences in single body composition variables among on-time, early, and late puberty groups. *P*-values for pairwise comparisons among three pubertal timing groups were adjusted using a Bonferroni correction. A multinomial logistic regression model was used to examine the association between body composition and pubertal timing, with on-time puberty as a reference. The IPW method based on propensity score was implemented to control for potential confounding. Firstly, propensity score estimations of body composition variables were calculated by linear regression model based on least-square estimation. Body composition variables were regressed in the propensity estimation model on the covariates that have been reported as potential confounding factors, including age, family income, parental education level, physical activity, sedentary time, sleep duration, intake frequencies for meat, aquatic products, vegetables, bean products, fruits, egg and milk, and FM (when analyzing of FFM variables) or FFM (when analyzing of FM variables). Then, the weight of 1/propensity score was adopted for the sample weighting process. The correlation coefficient was calculated to evaluate the quality of balancing performance after the weighting process. After that, the association between body composition and puberty timing was investigated by a multinomial logistic regression model within the weighted sample. Differences between OR*s* were compared by the *Z* test. To assess the sensitivity of results for the choice of statistical models and acquire robust effect estimations, traditional covariate adjustment regression analysis was performed, and the corresponding results were compared with IPW regression results. All reported *P*-values were two-sided, with *P* < 0.05 as statistically significant.

## Results

A total of 1,493 boys and 1,261 girls who had entered puberty were finally included in the analysis. Table 1 shows the characteristics of participants by gender. Boys had an average age of 12.81 (standard deviation 1.49) years and girls of 12.57 (standard deviation 1.57) years. According to the quantiles of age of each Tanner stage, 373 (24.98%) boys and 303 (24.03%) girls were assigned to early puberty, 738 (49.43%) boys and 641 (50.83%) girls to on-time puberty, and 382 (25.59%) boys and 317 (25.14%) girls to late puberty.

**Table 1:** Characteristics of participants in Tanner stage II–V.

**Variables**	**Boys *N* = 1,493**	**Girls *N* = 1,261**
Age, mean ± SD	12.81 ± 1.49	12.57 ± 1.57
**Father's educational level**, ***n*** **(%)**		
Junior high school education or lower	435 (29.14)	329 (26.09)
Senior high school education	369 (24.72)	269 (21.33)
Junior college or higher	689 (46.14)	663 (52.58)
**Mother's educational level**, ***n*** **(%)**		
Junior high school education or lower	547 (36.64)	382 (30.29)
Senior high school education	311 (20.83)	277 (21.97)
Junior college or higher	635 (42.53)	602 (47.74)
**Family income (yuan/ year)**, ***n*** **(%)**		
<100,000	385 (25.79)	319 (25.30)
100,000–200,000	505 (33.82)	389 (30.85)
≥200,000	603 (40.39)	553 (43.85)
**Pubertal timing**, ***n*** **(%)**		
On-time	738 (49.43)	641 (50.83)
Early	373 (24.98)	303 (24.03)
Late	382 (25.59)	317 (25.14)
**Tanner stages**, ***n*** **(%)**		
G2/B2	412 (27.60)	461 (36.56)
G3/B3	652 (43.67)	538 (42.66)
G4/B4	407 (27.26)	230 (18.24)
G5/B5	22 (1.47)	32 (2.54)
Sedentary time (h/d), mean ± SD	3.91 ± 2.53	4.08 ± 2.45
Physical activity (h/d), mean ± SD	0.96 ± 0.98	0.76 ± 0.82
Sleep duration (h/d), mean ± SD	9.42 ± 1.61	9.17 ± 1.27
Frequency for Meat (times/week), median (IQR)	5 (4, 6)	5 (4, 6)
Frequency for aquatic products (times/week), median (IQR)	3 (3, 4)	3 (3, 4)
Frequency for egg (times/week), median (IQR)	4 (3, 4)	4 (3, 4)
Frequency for bean products (times/week), median (IQR)	3 (3, 4)	3 (3, 4)
Frequency for fruits (times/week), median (IQR)	4 (4, 5)	4 (4, 5)
Frequency for vegetables (times/week), median (IQR)	4 (4, 5)	5 (4, 6)
Frequency for milk (times/week), median (IQR)	3 (3, 3)	3 (3, 3)
BF%, mean ± SD	16.9 ± 8.6	24.3 ± 7.0
BF% *Z*-score, mean ± SD	−0.00 ± 0.98	0.07 ± 1.00
FMI, mean ± SD	3.6 ± 2.5	4.8 ± 2.2
FMI Z-score, mean ± SD	0.00 ± 0.98	0.07 ± 1.01
FFM%, mean ± SD	83.1 ± 8.6	75.8 ± 7.0
FFM% *Z*-score, mean ± SD	0.01 ± 0.98	−0.07 ± 1.00
FFMI, mean ± SD	16.0 ± 1.9	14.3 ± 1.2
FFMI *Z*-score, mean ± SD	0.06 ± 0.99	0.10 ± 1.00
ASMI, mean ± SD	6.4 ± 1.1	5.4 ± 0.7
ASMI *Z*-score, mean ± SD	0.07 ± 0.99	0.10 ± 1.00

*BF%, body fat percentage; FMI, fat mass index; FFM%, fat-free mass percentage; FFMI, fat-free mass index; ASMI, appendicular skeletal mass index; SD, standard deviation; IQR, interquartile range*.

*Values are mean ± standard deviation (SD), median (lower quartile, upper quartile) or frequency (percentage) as appropriate*.

As compared to those with on-time puberty, boys with late puberty had lower Z-scores of FFMI, and ASMI (*P* for Bonferroni = 0.018, and 0.003, respectively), and boys with early puberty appeared to have lower BF% *Z*-score and higher FFM% *Z*-score (*P* for Bonferroni = 0.063 and 0.065, respectively). Non-significant differences were observed in other body composition *Z*-scores between boys with on-time and late puberty or early puberty. For girls, when compared to on-time pubertal peers, those with early puberty had higher *Z*-scores of BF%, FMI, FFMI, and ASMI but lower *Z*-score of FFM% (all *P* for Bonferroni < 0.001, Table 2), while girls in late puberty showed no different *Z*-scores of body composition (all *P* > 0.05).

**Table 2:** Body composition characteristics in boys and girls with early, on-time, and late puberty.

**Variables**	**On-time**	**Early**	**Late**	***t* ^a^**	***P* ^a^**	***t* ^b^**	***P* ^b^**
Boys	*n* = 738	*n* = 373	*n* = 382				
BF%	17.3 ± 8.7	17.2 ± 8.3	15.9 ± 8.8	0.33	0.760	2.62	**0.021**
BF% *Z*-score	0.01 ± 0.95	−0.12 ± 0.91	0.08 ± 1.09	2.18	0.063	−1.01	0.359
FMI	3.7 ± 2.5	3.4 ± 2.3	3.5 ± 2.7	1.53	0.218	1.28	0.254
FMI *Z*-score	0.01 ± 0.94	−0.09 ± 0.92	0.07 ± 1.10	1.77	0.148	−0.90	0.410
FFM%	82.7 ± 8.7	82.8 ± 8.3	84.1 ± 8.8	−0.30	0.761	−2.63	**0.021**
FFM% *Z*-score	−0.01 ± 0.95	0.12 ± 0.91	−0.07 ± 1.09	−2.16	0.065	1.01	0.359
FFMI	16.0 ± 1.9	15.3 ± 1.8	16.5 ± 1.8	6.46	**<0.001**	−3.89	**<0.001**
FFMI *Z*-score	0.08 ± 0.97	0.17 ± 0.99	−0.09 ± 1.02	−1.37	0.243	2.71	**0.018**
ASMI	6.5 ± 1.1	6.0 ± 1.1	6.8 ± 0.9	7.48	**<0.001**	−5.80	**<0.001**
ASMI *Z*-score	0.09 ± 0.96	0.20 ± 0.96	−0.12 ± 1.03	−1.79	0.146	3.30	**0.003**
Girls	*n* = 641	*n* = 303	*n* = 317				
BF%	24.3 ± 7.1	23.6 ± 7.6	24.9 ± 6.2	1.35	0.243	−1.42	0.243
BF% *Z*-score	0.00 ± 0.98	0.36 ± 1.00	−0.07 ± 0.98	−5.20	**<0.001**	1.08	0.335
FMI	4.9 ± 2.2	4.6 ± 2.3	4.9 ± 1.9	1.59	0.209	−0.64	0.555
FMI *Z*-score	0.01 ± 0.99	0.37 ± 1.07	−0.08 ± 0.94	−5.03	**<0.001**	1.28	0.254
FFM%	75.7 ± 7.1	76.4 ± 7.6	75.1 ± 6.2	−1.37	0.243	1.40	0.243
FFM% *Z*-score	0.00 ± 0.98	−0.36 ± 1.00	0.07 ± 0.98	5.19	**<0.001**	−1.10	0.335
FFMI	14.3 ± 1.3	14.0 ± 1.2	14.4 ± 1.1	3.48	**0.003**	−0.49	0.655
FFMI *Z*-score	0.03 ± 0.99	0.41 ± 0.98	−0.07 ± 0.97	−5.50	**<0.001**	1.55	0.215
ASMI	5.4 ± 0.7	5.0 ± 0.8	5.6 ± 0.5	7.46	**<0.001**	−3.24	**0.003**
ASMI *Z*-score	0.02 ± 0.99	0.41 ± 0.98	−0.03 ± 0.97	−5.69	**<0.001**	0.77	0.483

*BF%, body fat percentage; FMI, fat mass index; FFM%, fat-free mass percentage; FFMI, fat-free mass index; ASMI, appendicular skeletal mass index*.

*All P-values were Bonferroni-corrected*.

a *Early puberty vs. on-time puberty*.

b *Late puberty vs. on-time puberty*.

*Bold values indicates P < 0.05*.

After weighting the participants with inverse probability based on the propensity score, all covariates showed weak correlation coefficients with single body composition variables both in boys and girls (Supplementary Tables 1, 2), suggesting a good balance of participants between groups. In IPW regression analysis of boys, higher *Z*-scores of BF% and FMI were both significantly associated with the decreased risk of early puberty (OR = 0.75, 95% CI = 0.64–0.87, *P* < 0.001; OR = 0.74, 95% CI = 0.63–0.88, *P* = 0.001) but with the increased risk of late puberty (OR = 1.28, 95% CI = 1.06–1.55, *P* = 0.011; OR = 1.26, 95% CI = 1.02–1.56, *P* = 0.032). For the ASMI, increasing *Z*-score yielded significant associations with greater early puberty risk (OR = 1.26, 95% CI = 1.07–1.49, *P* = 0.007) but lower risk of late puberty (OR = 0.82, 95% CI = 0.69–0.99, *P* = 0.039). As for girls, in contrast to boys, higher *Z*-scores of BF% and FMI significantly increased the risk of early puberty onset (OR = 1.39, 95% CI = 1.19–1.64, *P* < 0.001; OR = 1.59, 95% CI = 1.33–1.90, *P* < 0.001, Table 3). Nevertheless, the associations of *Z*-scores of FFM variables with early puberty did not reach statistical significance, as well as FM and FFM *Z*-scores with late puberty. Additionally, significant differences in OR*s* for the association of FM with early puberty were seen between genders (*Z* = −5.45 and −6.24 for BF% and FMI, respectively; both *P* < 0.05). In sensitivity analysis, we examined the robustness of the body composition-puberty associations by traditional covariate adjustment regression. The traditional adjustment model retained the significant associations shown by the IPW regression analysis, but with wider confidence intervals for ORs (Supplementary Table 3).

**Table 3:** Associations of body composition with pubertal timing in boys and girls.

**Variables**	**Early vs. on-time puberty**				**Late vs. on-time puberty**			
	**Model 1**		**Model 2**		**Model 1**		**Model 2**	
	**OR (95% CI)**	***P***	**OR (95% CI)**	***P***	**OR (95% CI)**	***P***	**OR (95% CI)**	***P***
**Boys**								
BF% *Z*-score	0.86 (0.75–0.99)	**0.032**	0.75 (0.64–0.87)	**<0.001**	1.07 (0.94–1.21)	0.292	1.28 (1.06–1.55)	**0.011**
FMI *Z*-score	0.88 (0.77–1.01)	0.080	0.74 (0.63–0.88)	**0.001**	1.06 (0.94–1.20)	0.344	1.26 (1.02–1.56)	**0.032**
FFM% *Z*-score	1.16 (1.01–1.33)	**0.034**	0.91 (0.72–1.14)	0.407	0.94 (0.83–1.06)	0.292	0.81 (0.65–1.02)	0.069
FFMI *Z*-score	1.09 (0.96–1.24)	0.168	1.17 (0.99–1.40)	0.069	0.84 (0.74–0.95)	**0.006**	0.86 (0.70–1.05)	0.131
ASMI *Z*-score	1.13 (0.99–1.28)	0.073	1.26 (1.07–1.49)	**0.007**	0.81 (0.71–0.91)	**0.001**	0.82 (0.69–0.99)	**0.039**
**Girls**								
BF% *Z*-score	1.44 (1.25–1.65)	**<0.001**	1.39 (1.19–1.64)	**<0.001**	0.93 (0.81–1.06)	0.279	1.13 (0.91–1.41)	0.268
FMI *Z*-score	1.40 (1.23–1.60)	**<0.001**	1.59 (1.33–1.90)	**<0.001**	0.91 (0.79–1.05)	0.208	1.19 (0.95–1.48)	0.129
FFM% *Z*-score	0.70 (0.61–0.80)	**<0.001**	0.82 (0.64–1.05)	0.123	1.08 (0.94–1.24)	0.272	0.97 (0.77–1.23)	0.797
FFMI *Z*-score	1.46 (1.27–1.68)	**<0.001**	1.12 (0.88–1.44)	0.361	0.90 (0.78–1.03)	0.124	1.05 (0.83–1.33)	0.690
ASMI *Z*-score	1.48 (1.29–1.71)	**<0.001**	1.12 (0.87–1.44)	0.395	0.95 (0.83–1.09)	0.446	1.27 (0.97–1.65)	0.077

*BF%, body fat percentage; FMI, fat mass index; FFM%, fat-free mass percentage; FFMI, fat-free mass index; ASMI, appendicular skeletal mass index; OR, odds ratio; CI, Confidence interval*.

*Model 1: multinomial logistic regression model, unadjusted*.

*Model 2: multinomial logistic regression model, with propensity score-based inverse probability weighting. Bold values indicates P < 0.05*.

## Discussion

This cross-sectional study, consisting of 1,493 boys and 1,261 girls who have entered puberty, investigated the relationship between body composition and pubertal timing using the propensity score-based IPW method, which could produce less-biased effect estimates compared to the traditional adjustment method (40). We found that FM was closely associated with pubertal timing in both boys and girls, but the association differed. There was a positive association of increased FM with a high risk of early puberty in girls, but an inverse one in boys. Besides, FFM seemed to promote boys to early onset of puberty. Our findings can contribute to a better understanding of the relationship between body composition and pubertal timing and shed light on some important gender differences conferring pubertal development.

The connection between adiposity and early puberty has been already consistently reported in girls (35, 49–52), whichever measure of pubertal timing was used. We found a similarity to previous works in all our regression models. Puberty is regulated by the hypothalamic-pituitary-gonadal axis. Several biological mechanisms have been hypothesized to explain adiposity-promoting gonadal axis initiation. One of the hypotheses is that increased adiposity could enhance aromatase activity and stimulate the conversion of androgens into estrogen, thereby triggering earlier pubertal onset (53). Similarly, insulin resistance induced by adiposity is linked to a decreased level of sex-hormone-binding globulin, which increases sex steroid bioavailability and precipitates early breast development (54). Besides, over-expressed leptin due to increased FM may also drive the central pulsatile release of gonadotrophin and accelerate the initiation of puberty (55). Nevertheless, the data linking adiposity to puberty in boys is even more limited and reveals a lack of concurrence. In this study, both traditional adjustment and IPW analyses that showed increased BF% and FMI were inversely associated with early puberty risk but positively associated with late puberty risk, consistent with the previous work on NHANES III data (56). Our findings add to the small body of literature that the pattern in the relationship of FM with pubertal timing differed by gender. Although the behind mechanism is unexplained, some lines of evidence now support our findings. Increased aromatization of androgens to estrogens driven by high adiposity can subsequently in turn inhibit gonadotropin secretion, which is related to a form of hypogonadotropic hypogonadism in males (57). An additional explanation may be related to gender divergence in leptin, an initial contributor to facilitate puberty onset. With the pubertal onset, leptin levels increased in girls but decreased in boys (58). Between leptin and FM, there was a positive association in girls but a non-significant one in boys (59). Meanwhile, leptin is obviously associated with early puberty in girls, but not in boys, or even possibly associated with delayed puberty in boys. One relevant observation is the positive correlation of leptin level with estradiol in girls but a negative one with testosterone in boys (60). On balance, there seemed to be biological plausibility with respect to the reverse relationship between FM and pubertal timing for boys in contrast to girls.

It is worth noting that a negative relationship between leptin level with FFM has been observed in boys, but a positive one in girls (59). Data related to gender differences in the accumulation of body composition have shown that boys gained more FFM while girls gained more FM (32). Intriguingly, we found a positive association of ASMI with early puberty and a negative association with late puberty in boys but a null association in girls in IPW analysis. Based on the aforementioned evidence, the gender differences in leptin involved in puberty may provide an explanation as to why high FFM appeared to advance the onset of puberty in boys. However, to our knowledge, only two of the previous studies have explored the relationship between FFM and pubertal timing in boys, one cross-sectional study in adolescents of Chongqing, China, and the other, a longitudinal study in children of Taiwan, China (35, 49). The former indicated a null association, while the latter suggested a positive association. More research will be needed to better understand the relationship of body composition with boys' pubertal timing and the underlying mechanism.

This study has several key strengths. A unique strength is that we utilized the IPW method based on propensity score to equate the confounding between pubertal groups and thereby can acquire robust results. In the analytical process, we further implemented the traditional adjustment model as sensitivity analysis, which reported a similar pattern of results to IPW analysis. Additionally, the results from the IPW method showed narrower 95% confidence intervals of effect estimates compared to the traditional adjustment method, further confirming the robustness of our findings. Furthermore, direct examination of breast development and testicular volume by the same well-trained operators allowed us to avoid a recall and reporting bias for assessing the pubertal stage.

Our findings should be interpreted in the context of the following limitations. One limitation concerns the cross-sectional nature, and thereby this study can only establish an association, but not causation. Increasing evidence appears to suggest a bidirectional causal relationship between adiposity and pubertal onset in girls (49, 61, 62). For this consideration, pubertal timing may also influence FFM accumulation. The causal direction between body composition and pubertal timing remains uncertain. Second, although we have controlled for known and measured confounders using the IPW approach, confounding by unmeasured or unknown variables can never be ruled out. Third, although BIA showed good consistency with DXA for measuring FFM, it might underestimate FM (63). Accordingly, the strength of FM-puberty associations might be underestimated in our present study. Nevertheless, the direction of the association should be reliable. Additionally, the study populations were only based in the Guangzhou region, China. Our findings may not be generalizable to other regions of China or other countries, due to ethnicity sensitivity concerning body composition and pubertal timing.

In sum, this study provides some novel contributions to the debate on the relationship between body composition and pubertal timing *via* the innovative methodology. Propensity score-based IPW analysis suggests gender dimorphisms in the connection of body composition with pubertal timing. While girls have a positive association of FM with early puberty, in boys the association is negative with early puberty and positive with late puberty. By contrast, FFM appears to be positively associated with early puberty and negatively associated with late puberty in boys, but not in girls. Future longitudinal studies or bidirectional Mendelian randomization analyses with large sample sizes are still needed to clarify the causal direction in the connection of body composition with pubertal onset.

## Data availability statement

The raw data supporting the conclusions of this article will be made available by the authors, without undue reservation.

## Ethics statement

The studies involving human participants were reviewed and approved by the Ethics Review Committee of Guangdong Pharmaceutical University, China (Medical Ethics Review No.: 2018-27). Written informed consent to participate in this study was provided by the participants' legal guardian/next of kin.

## Author contributions

YZ: methodology, investigation, data curation, and writing—original draft. JL: investigation and resources. DZ, LY, SL, and WY: investigation and data curation. WT: resources and supervision. YY: conceptualization, resources, supervision, and project administration. LL: conceptualization, methodology, investigation, resources, writing—review and editing, supervision, project administration, and funding acquisition. All authors contributed to the article and approved the submitted version.

## Funding

This work was supported by the National Natural Science Foundation of China (81874271).

## Conflict of interest

The authors declare that the research was conducted in the absence of any commercial or financial relationships that could be construed as a potential conflict of interest.

## Publisher's note

All claims expressed in this article are solely those of the authors and do not necessarily represent those of their affiliated organizations, or those of the publisher, the editors and the reviewers. Any product that may be evaluated in this article, or claim that may be made by its manufacturer, is not guaranteed or endorsed by the publisher.
