# Supplementary material for: Association of body composition with pubertal timing in children and adolescents from Guangzhou, China
Source: Front Public Health. 2022 Aug 17;10:943886. doi: 10.3389/fpubh.2022.943886 (PMC9428289; doi:10.3389/fpubh.2022.943886)
Supplement: Supplementary file 1 [file Data_Sheet_1.docx]

**S-Table 1. Correlation coefficient of Z-score of body composition variables with covariate in boys in original and weighted sample.**

| Variables | BF%  Z-score | FMI  Z-score | FFM%  Z-score | FFMI  Z-score | ASMI  Z-score |
| --- | --- | --- | --- | --- | --- |
| Original sample |  |  |  |  |  |
| Age | -0.06 | -0.05 | 0.06 | -0.04 | -0.04 |
| Father's educational level | 0.03 | 0.03 | -0.03 | 0.05 | 0.06 |
| Mather's educational level | 0.05 | 0.04 | -0.05 | 0.05 | 0.06 |
| Family income | 0.06 | 0.06 | -0.06 | 0.06 | 0.07 |
| Sedentary behavior | -0.04 | -0.03 | 0.04 | 0.04 | 0.06 |
| Physical activity | -0.05 | -0.04 | 0.05 | 0.05 | 0.04 |
| Sleep duration | -0.01 | -0.02 | 0.01 | -0.05 | -0.06 |
| Frequency for Meat | 0.03 | 0.03 | -0.03 | 0.02 | 0.04 |
| Frequency for aquatic products | -0.03 | -0.03 | 0.03 | 0.01 | 0.01 |
| Frequency for egg | 0.03 | 0.02 | -0.03 | 0.05 | 0.05 |
| Frequency for bean Products | 0.03 | 0.03 | -0.03 | -0.01 | 0.00 |
| Frequency for fruits | 0.04 | 0.05 | -0.04 | 0.06 | 0.05 |
| Frequency for vegetables | 0.03 | 0.03 | -0.03 | 0.04 | 0.03 |
| Frequency for milk | 0.02 | 0.02 | -0.02 | 0.01 | 0.01 |
| Mean of correlation coefficient^*^ | 0.04 | 0.03 | 0.04 | 0.04 | 0.04 |
| Weighted sample |  |  |  |  |  |
| Age | 0.04 | 0.03 | 0.11 | -0.02 | -0.02 |
| Father's educational level | -0.04 | -0.02 | 0.08 | 0.02 | 0.00 |
| Mather's educational level | -0.05 | -0.05 | 0.01 | 0.04 | 0.02 |
| Family income | -0.03 | -0.03 | -0.02 | 0.03 | 0.02 |
| Sedentary behavior | 0.00 | -0.01 | 0.02 | 0.05 | 0.02 |
| Physical activity | 0.03 | 0.03 | 0.05 | -0.01 | 0.00 |
| Sleep duration | -0.01 | -0.01 | 0.02 | 0.00 | 0.04 |
| Frequency for Meat | 0.03 | 0.04 | -0.03 | 0.04 | 0.02 |
| Frequency for aquatic products | 0.03 | 0.02 | 0.05 | 0.01 | 0.02 |
| Frequency for egg | -0.03 | -0.01 | 0.04 | 0.03 | 0.04 |
| Frequency for bean Products | -0.04 | -0.06 | -0.01 | 0.02 | 0.02 |
| Frequency for fruits | -0.02 | -0.02 | -0.04 | 0.06 | 0.03 |
| Frequency for vegetables | 0.03 | 0.03 | -0.01 | 0.03 | 0.00 |
| Frequency for milk | -0.06 | -0.05 | -0.05 | 0.03 | 0.03 |
| Mean of Correlation coefficient^*^ | 0.03 | 0.03 | 0.04 | 0.03 | 0.02 |

Abbreviations: BF%, body fat percentage; FMI, fat mass index; FFM%, fat-free mass percentage; FFMI, fat-free mass index; ASMI, appendicular skeletal mass index.

^*^A series of correlation coefficients of a body composition variable with covariates were converted into absolute value, and then its average value was calculated.

**S-Table 2. Correlation coefficients of Z-scores of body composition variables with covariate after weighting in girls in original and weighted sample.**

| Variables | BF%  Z-score | FMI  Z-score | FFM%  Z-score | FFMI  Z-score | ASMI  Z-score |
| --- | --- | --- | --- | --- | --- |
| Original sample |  |  |  |  |  |
| Age | -0.08 | -0.08 | 0.08 | -0.12 | -0.13 |
| Father's educational level | 0.04 | 0.04 | -0.04 | 0.01 | 0.04 |
| Mather's educational level | 0.09 | 0.07 | -0.09 | 0.04 | 0.08 |
| Family income | 0.06 | 0.05 | -0.06 | 0.03 | 0.08 |
| Sedentary behavior | -0.04 | -0.03 | 0.04 | 0.04 | 0.06 |
| Physical activity | -0.05 | -0.04 | 0.05 | 0.05 | 0.04 |
| Sleep duration | -0.01 | -0.02 | 0.01 | -0.05 | -0.06 |
| Frequency for Meat | 0.01 | 0.00 | -0.01 | 0.00 | -0.01 |
| Frequency for aquatic products | -0.03 | -0.03 | 0.03 | -0.02 | -0.04 |
| Frequency for egg | 0.06 | 0.05 | -0.06 | 0.02 | 0.01 |
| Frequency for bean Products | -0.02 | -0.03 | 0.02 | -0.01 | -0.02 |
| Frequency for fruits | -0.02 | 0.00 | 0.02 | 0.05 | 0.07 |
| Frequency for vegetables | 0.04 | 0.05 | -0.04 | 0.05 | 0.04 |
| Frequency for milk | 0.00 | -0.01 | 0.00 | -0.03 | -0.02 |
| Mean of correlation coefficient^*^ | 0.04 | 0.04 | 0.04 | 0.04 | 0.05 |
| Weighted sample |  |  |  |  |  |
| Age | -0.03 | -0.13 | 0.03 | 0.02 | 0.04 |
| Father's educational level | 0.02 | 0.03 | -0.07 | 0.02 | 0.03 |
| Mather's educational level | 0.01 | 0.04 | -0.05 | 0.01 | 0.03 |
| Family income | 0.00 | 0.01 | -0.03 | -0.01 | 0.02 |
| Sedentary behavior | 0.01 | 0.01 | -0.11 | -0.02 | -0.04 |
| Physical activity | 0.00 | -0.02 | 0.00 | 0.03 | 0.01 |
| Sleep duration | 0.00 | 0.03 | 0.06 | -0.06 | -0.06 |
| Frequency for Meat | -0.03 | -0.03 | -0.02 | 0.00 | 0.06 |
| Frequency for aquatic products | -0.03 | -0.06 | -0.04 | 0.00 | -0.01 |
| Frequency for egg | 0.00 | 0.02 | 0.00 | -0.03 | -0.01 |
| Frequency for bean Products | -0.01 | 0.00 | 0.07 | -0.03 | -0.04 |
| Frequency for fruits | -0.02 | 0.00 | -0.02 | -0.04 | -0.04 |
| Frequency for vegetables | -0.02 | -0.03 | -0.06 | -0.01 | 0.02 |
| Frequency for milk | 0.07 | 0.09 | -0.05 | -0.05 | -0.06 |
| Mean of Correlation coefficient^*^ | 0.02 | 0.04 | 0.04 | 0.02 | 0.03 |

Abbreviations: BF%, body fat percentage; FMI, fat mass index; FFM%, fat-free mass percentage; FFMI, fat-free mass index; ASMI, appendicular skeletal mass index.

^*^A series of correlation coefficients of a body composition variable with covariates were converted into absolute value, and then its average value was calculated.

**S-Table 3. Association of body composition with pubertal timing** **using traditional logistic regression analysis with covariate adjustment in boys and girls.**

| Variables | Early VS. on-time puberty | |  | Late VS. on-time puberty | |
| --- | --- | --- | --- | --- | --- |
|  | *OR (95% CI)* | *P* |  | *OR (95% CI)* | *P* |
| Boys |  |  |  |  |  |
| BF% Z-score | 0.65 (0.55-0.77) | **<0.001** |  | 1.88 (1.54-2.31) | **<0.001** |
| FMI Z-score | 0.65 (0.54-0.78) | **<0.001** |  | 1.99 (1.62-2.46) | **<0.001** |
| FFM% Z-score | 2.20 (1.40-3.48) | **<0.001** |  | 0.21 (0.11-0.37) | **<0.001** |
| FFMI Z-score | 1.36 (1.14-1.63) | **<0.001** |  | 0.42 (0.33-0.53) | **<0.001** |
| ASMI Z-score | 1.40 (1.18-1.68) | **<0.001** |  | 0.39 (0.31-0.49) | **<0.001** |
| Girls |  |  |  |  |  |
| BF% Z-score | 1.38 (1.13-1.70) | **0.002** |  | 0.67 (0.53-0.84) | **0.001** |
| FMI Z-score | 1.25 (1.02-1.53) | **0.030** |  | 0.65 (0.50-0.83) | **0.001** |
| FFM% Z-score | 1.13 (0.68-1.86) | 0.645 |  | 2.16 (1.09-4.28) | **0.027** |
| FFMI Z-score | 1.02 (0.80-1.29) | 0.880 |  | 0.83 (0.64-1.07) | 0.147 |
| ASMI Z-score | 0.95 (0.74-1.21) | 0.663 |  | 0.99 (0.76-1.29) | 0.947 |

Abbreviations: BF%, body fat percentage; FMI, fat mass index; FFM%, fat-free mass percentage; FFMI, fat-free mass index; ASMI, appendicular skeletal mass index; OR, odds ratio; CI, Confidence interval.

Models adjusted for age, family income, father’s educational level, mother’s educational level, physical exercise time, sedentary behavior time, sleep duration, intake frequency for meat, aquatic products, vegetables, bean products, fruits, egg and milk, and FM (in analysis of FFM variables) or FFM (in analysis of FM variables).
